# Screening of Non- *Saccharomyces cerevisiae* Strains for Tolerance to Formic Acid in Bioethanol Fermentation

**DOI:** 10.1371/journal.pone.0135626

**Published:** 2015-08-18

**Authors:** Cyprian E. Oshoma, Darren Greetham, Edward J. Louis, Katherine A. Smart, Trevor G. Phister, Chris Powell, Chenyu Du

**Affiliations:** 1 Bioenergy and Brewing Science Building, School of Biosciences, University of Nottingham, Sutton Bonington Campus, Loughborough, Leics, United Kingdom; 2 Centre for Genetic Architecture of Complex Traits, University of Leicester, Leicester, United Kingdom; 3 SAB Miller PLC, Surrey, United Kingdom; 4 PepsiCo Int. Beaumont Park, Leycroft Road, Leicester, United Kingdom; 5 School of Applied Sciences, University of Huddersfield, Queensgate, Huddersfield, United Kingdom; National Renewable Energy Lab, UNITED STATES

## Abstract

Formic acid is one of the major inhibitory compounds present in hydrolysates derived from lignocellulosic materials, the presence of which can significantly hamper the efficiency of converting available sugars into bioethanol. This study investigated the potential for screening formic acid tolerance in non-*Saccharomyces cerevisiae* yeast strains, which could be used for the development of advanced generation bioethanol processes. Spot plate and phenotypic microarray methods were used to screen the formic acid tolerance of 7 non-*Saccharomyces cerevisiae* yeasts. *S*. *kudriavzeii* IFO1802 and *S*. *arboricolus* 2.3319 displayed a higher formic acid tolerance when compared to other strains in the study. Strain *S*. *arboricolus* 2.3319 was selected for further investigation due to its genetic variability among the *Saccharomyces* species as related to *Saccharomyces cerevisiae* and availability of two sibling strains: *S*. *arboricolus* 2.3317 and 2.3318 in the lab. The tolerance of *S*. *arboricolus* strains (2.3317, 2.3318 and 2.3319) to formic acid was further investigated by lab-scale fermentation analysis, and compared with *S*. *cerevisiae* NCYC2592. *S*. *arboricolus* 2.3319 demonstrated improved formic acid tolerance and a similar bioethanol synthesis capacity to *S*. *cerevisiae* NCYC2592, while *S*. *arboricolus* 2.3317 and 2.3318 exhibited an overall inferior performance. Metabolite analysis indicated that *S*. *arboricolus* strain 2.3319 accumulated comparatively high concentrations of glycerol and glycogen, which may have contributed to its ability to tolerate high levels of formic acid.

## Introduction

The importance of identifying alternative energy sources has become necessary due to the continuous depletion of limited fossil fuel stock and for the creation of a safe and sustainable environment. Recently, attention has focused on renewable or alternative sources of energy, as a means of supplementing the inevitable shortage of world’s energy supply [1]. In some developing countries, there is a need for alternative sources of energy, such as those derived from lignocellulosic biomass including herbaceous and woody plants, agricultural, forestry residues, municipal solid waste and industrial waste streams [2, 3]. These feedstocks are of particular interest as they do not compete with food production for agricultural resources [4].

Lignocellulosic plant residues containing up to 70% carbohydrate (as cellulose and hemicellulose) are prominent substrates for the advanced generation of bioethanol production. However, due to the recalcitrant nature of lignocellulosic biomass, pretreatment is necessary for the release of fermentable sugars. Pretreatment processing can be carried out in different ways including mechanical, steam explosion, ammonia fiber explosion, acid or alkaline pretreatment and biological pretreatment [1]. Furthermore, a combination of two or more of these processes can be employed with a view to producing synergetic effects.

Rapid and efficient fermentation of lignocellulosic hydrolysates is limited because, in addition to the release of monomeric sugars, a range of inhibitory compounds are generated during pretreatment and hydrolysis [5, 6, 7, 8]. These inhibitory compounds fall into specific groups such as weak acids, furan derivatives and phenolic compounds [9]. The types of toxic compounds generated, and their concentrations in lignocellulosic hydrolysates, depend on both the raw material and the operational conditions employed for hydrolysis [10]. Toxic compounds can act to stress fermentative organisms to a point beyond which the efficient utilization of sugars is possible, ultimately leading to reduced product formation [11].

Formic acid is one of the weak acid inhibitors present in lignocellulosic hydrolysates, with a typical concentration of approximately 1.4 g/L (30 mM) [8, 12]. The inhibitory effect of formic acid has been ascribed to both uncoupling and intracellular anion accumulation [13, 14] and the reduction of the uptake of aromatic amino acids [12]. The undissociated form of weak acids can diffuse from the fermentation medium across the plasma membrane [13, 14] and dissociate due to higher intracellular pH, thus decreasing the cytosolic pH. The decrease in intracellular pH is compensated by the plasma membrane ATPase, which pumps protons out of the cell at the expense of ATP hydrolysis. Consequently, less ATP is available for biomass formation. According to the intracellular anion accumulation theory, the anionic form of the acid is captured inside the cell and the undissociated acid will diffuse out of the cell until equilibrium is reached. Weak acids have also been shown to inhibit yeast growth by reducing the uptake of aromatic amino acids from the medium, probably as a consequence of strong inhibition of the enzyme permease [12]. Formic acid is more toxic to yeast strains than either acetic acid or levulinic acid [12, 15], due to a lower pK_a_ value (3.75 at 20°C) than acetic (4.75 at 25°C) and levulinic acid (4.66 at 25°C). Its undissociated form should be found in lower concentrations at the same internal pH, and consequently be less toxic to the cells. The increased toxicity of formic acid seems to be associated with a smaller molecule size, which may facilitate its diffusion through the plasma membrane and possibly its higher anion toxicity [16].

Yeasts, mostly strains of *Saccharomyces cerevisiae*, have been widely used for bioethanol production industrially, due to their high fermentative ability, ethanol tolerance and rapid growth under anaerobic conditions [17]. These yeasts, however, are susceptible to inhibitory compounds present in lignocellulose derived hydrolysates [18]. One possible solution is to detoxify the hydrolysate to remove the inhibitors, however, this creates additional costs and a potential loss of sugar [12]. An alternative approach and long-term solution to overcome this problem is to either screen for high inhibitor tolerant yeast strains or create genetically modified strains with desired tolerance properties. Research in these areas has focused on *S*. *cerevisiae* strains while exploitation of alternative species for improved inhibitor tolerance has been limited. Wimalasena *et al*. [19] recently screened *Saccharomyces* spp. (previously termed Saccharomyces sensu stricto) for their tolerance to osmosis, temperature, ethanol and inhibitors using phenotypic microarray analysis. The results indicated that some non-*S*. *cerevisiae* yeast strains could have promising properties to be used in lignocellulosic bioethanol fermentation.

In this study, the screening of yeast strains other than *S*. *cerevisiae* for high formic acid tolerance was conducted. Selected high tolerance strains were investigated for ethanol fermentation under formic acid stress, compared to a typical reference *S*. *cerevisiae* strain. It is anticipated that the selected strains, with an innate tolerance to inhibitors, could lead to improved bioethanol production from lignocellulose.

## Materials and Methods

### Microorganisms

All the yeast strains used in this study are listed in Table 1. The strains were stored in glycerol stock at -80°C until required. The inoculum was prepared by taking a loop full of stock culture to 10 mL YPD (yeast extract 10 g/L, peptone 20 g/L and glucose 20 g/L) broth and incubating in 30 mL sterlin tube at 30°C, 120 rpm for 48 hours.

**Table 1:** Strain number and names of *Saccharomyces* spp. used in the study.

Strain	Culture Number	Organism
1	DBVPG6466	*Saccharomyces paradoxus*
2	IFO1816	*Saccharomyces mikatae*
3	CBS432	*Saccharomyces paradoxus*
4	DBVPG6299	*Saccharomyces bayanus*
5	2.3319	*Saccharomyces arboricolus*
6	IFO1802	*Saccharomyces kudriavzeii*
7	DBVPG6298	*Saccharomyces castelli*
8	NCYC2592	*Saccharomyces cerevisiae*
9	2.3317	*Saccharomyces arboricolus*
10	2.3318	*Saccharomyces arboricolus*

### Spot plate analysis

Spot plate tests were performed according to Homann *et al*. [20] with modifications. YPD agar (YPD plus No. 1 agar 15 g/L) incorporating varying concentrations of formic acid (0, 25, 30, 35 or 40 mM) was used to screen for the tolerance level of the various strains. Yeast Nitrogen Base (YNB) agar composed of YNB 6.7 g/L, glucose 20 g/L, No.1 agar 15 g/L. YNB agar with addition formic acid at concentrations of 0, 10, 15 20 or 25 mM was employed for tolerance level screening. The yeast culture was diluted to an OD of 1.0 (an estimated cell number of 10^7^ cells/mL) with distilled water. Then 5 μL samples of ten-fold serial dilution of the yeast cultures were spotted on YPD or YNB agar plates. The plates were incubated anaerobically at 30°C for 48 hours and growth differences were recorded photographically using a Bio-Rad-transilluminator (Bio-Rad, Cambridge, UK).

### Phenotype Microarray (PM) analysis

Biolog medium comprising YNB 6.7 g/L, glucose 60 g/L, nutrient supplement 2.6 μl/L (48x NS solution g/L: adenine HCl 4.12, L-histidine HCl monohydrate 1.01, L-leucine 6.3, L-lysine HCl 4.38, L-methionine 9.6, L-tryptophan 2.45 and Uracil 1.61) and a proprietary stain known as dye D 0.2 μL (Biolog, USA). Final volume of 30 μL was made up using distilled water and transferred into various wells with different concentrations of formic acid (0, 10, 15, 20 or 25 mM). Wells also contained 75 μL IFY buffer (Biolog, USA), 3.8 μL of yeast (previously adjusted to 62% transmittance) and 11.2 μL distilled water. 96-well plates were loaded into Omnilog reader (Biolog, USA) and incubated at 30°C for 96 hours under anaerobic condition; data was recorded photographically at 15-minute intervals. The conversion of dye intensity was detected and transformed into a signal value that reflected cell metabolic activity and dye conversion. The signal data were compiled upon completion of the incubation and data exported from the Biolog software into Microsoft Excel. Each experiment was performed in triplicate.

### Culture propagation for laboratory scale fermentations

*S*. *arboricolus* 2.3317, 2.3318, 2.3319 and *S*. *cerevisiae* NCYC2592 were employed in lab scale fermentations to investigate formic acid tolerance. A loop full of strain stock was aseptically inoculated into 10 mL of YPD broth and incubated at 30°C for 48 hours and at 120 rpm. The 10 mL culture was transferred into 100 ml of YPD broth and cultured for 48 hours at 30°C, 120 rpm, and the whole culture was finally transferred into 1 L of YPD and cultured for 48 hours at 30°C and 120 rpm. The 1 L culture was centrifuged at 5000 rpm at 4°C for 5 minutes. The supernatant was discarded and the wet pellet was used for inoculation.

### Fermentation with addition of formic acid

YPD broth with the addition of formic acid at 0, 10, 20, 30, 40, 50 or 60 mM was used in the laboratory scale fermentations. The pH of the media was adjusted to 4.5 using phosphoric acid and/or NaOH under aseptic conditions. From the broth, 100 mL was transferred into mini fermentation vessels (FVs). The prepared 0.4 g (wet weight) of yeast pellet was aseptically transferred into each of the bottles. Then the bottle was sealed and equipped with a bubbling CO_2_ outlet. All bottles were incubated at 30°C with shaking at 200 rpm for 24 hours. Samples were collected at specific time intervals to determine the total cell count, and concentrations of glucose, ethanol, glycerol and glycogen. All fermentations were carried out in triplicate.

### Total cell number analysis

The total cell number was determined with a haemocytometer according to the method of Sami *et al*. [21]. Methylene blue 0.01% (w/v) was dissolved in sodium citrate 2% (w/v) solution. Yeast broth was diluted using sterile water. The cell suspension was mixed with methylene blue solution in a ratio of 1:1. The solution was examined microscopically and total cell number was counted.

### HPLC analysis

Glucose, ethanol, and glycerol concentrations were determined using a JASCO HPLC system composed of a JASCO AS-2055 Intelligent Autosampler (JASCO, Essex, UK), and a JASCO PU-1580 Intelligent HPLC pump. The Rezex ROA organic acid H^+^ organic acid column (5μm, 7.8mm × 300mm, Phenomenex, Macclesfield, UK) was used and the mobile phase was 0.005 N H_2_SO_4_ with a flow rate 0.5 mL/minute.

### Intracellular glycogen analysis

The procedure of Parrou and Francois [22] was employed for the determination of intracellular glycogen. Total cells of 1 × 10^9^ cell/mL were obtained from the culture, centrifuge at 3,500 rpm for 5 min at 4°C and pellet was washed three times with distilled water. The washed cell pellet was lysed in 250 μL of sodium carbonate (0.25 M) incubated for 2 hours in a 95°C water bath with occasional stirring. To the cell suspension, 600 μL of sodium acetate (0.2 M) and 150 μL of acetic acid (1 M) were added respectively. From the cell suspension, 500 μL was transferred into a fresh Eppendorf tube and 10uL of α-amyloglucosidase (10 mg/mL; Sigma-Aldrich) was added and incubated at a 57°C for 12 hours in water bath. After overnight incubation, samples were centrifuged at 11,000×g for 2 minutes. The liberated glucose was quantified using a glucose assay kit (GOPOD; Megazyme). Analyses were carried out in triplicate and results expressed in glucose concentration as a function of cell number.

## Results

### Strain screening using spot plate assay

**Fig 1:**
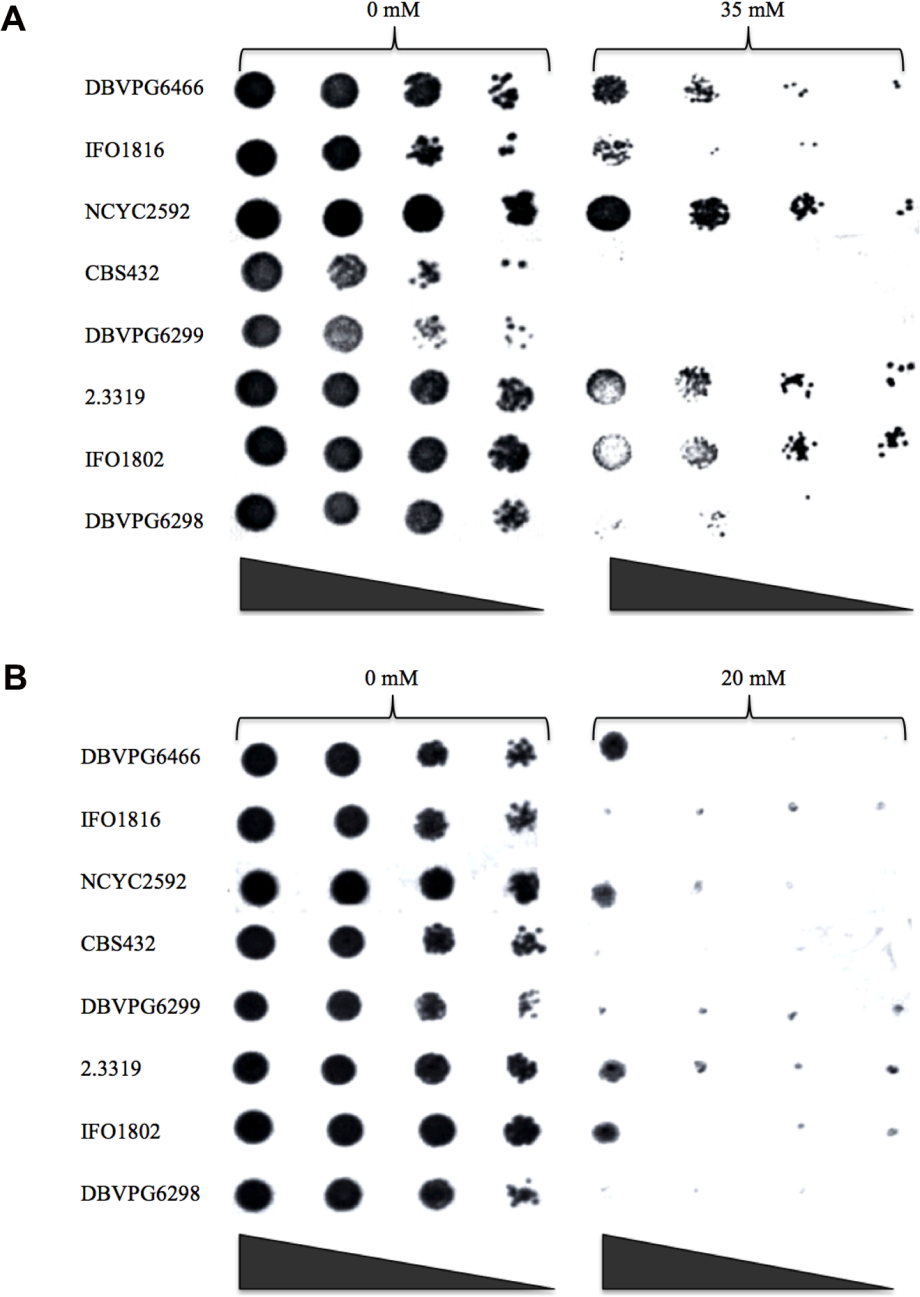
Tolerance to formic acid of *Saccharomyces* spp on solid medium using (A) YPD media with formic acid 0 and 35 mM; (B) YNB media with formic acid 0 and 20 mM. Aliquots 5 μL from tenfold serial dilution yeast cultures (initially suspended to an OD of 1.0 with an estimated cell number of 10^7^ cells/mL) were spotted on the plates. Plates were incubated at 30°C under anaerobic conditions for 48 hours. The last concentration of formic acid that cell growth can be observed was defined as tolerance level.

Seven non-*S*. *cerevisiae* strains listed in Table 1 (strain number 1 to 7) were screened for their tolerance to formic acid alongside a typical lab *S*. *cerevisiae* strain (NCYC2592), which has been used in our lab previously for ethanol tolerance analysis [8]. In this study, the concentration of formic acid in the media ranged from 0 to 40 mM, which corresponds to the formic acid concentrations typically reported in pretreated biomass hydrolysates [8, 12]. The spot plate results demonstrated that all strains were able to grow on YPD and YNB under control conditions (Fig 1A and 1B and Table 2). Cell growth could be seen on all YPD plates when formic acid was present at concentrations of 25 mM or lower (data not shown). Although the presence of 35 mM formic acid prevented cell growth on most strains (Fig 1A), growth was observed for strains *S*. *paradoxus* DBVPG6466, *S*. *kudriavzeii* IFO1802, *S*. *arboricolus* 2.3319 and *S*. *cerevisiae* NCYC2592. At a concentration of 40 mM, no cell growth was observed for any of the strains analyzed (data not shown). In assays using YNB medium, all strains displayed a lower tolerance to formic acid. Strains *S*. *paradoxus* DBVPG6466, *S*. *kudriavzeii* IFO1802, *S*. *arboricolus* 2.3319 and *S*. *cerevisiae* NCYC2592 were only tolerant to 20 mM formic acid on YNB medium plates (Fig 1B, Table 2), suggesting that YPD as an enriched medium may have a higher buffering capacity than YNB, a minimal medium. The formic acid critical concentrations for these strains were summarized in Table 2.

**Table 2:** Summary of formic acid tolerance concentrations of *Saccharomyces* spp by spot plate analysis.

Strain	Culture Number	Formic acid concentration (mM)	
		YPD	YNB
1	DBVPG6466	35	20
2	IFO1816	30	15
3	CBS432	30	10
4	DBVPG6299	25	10
5	2.3319	35	20
6	IFO1802	35	20
7	DBVPG6298	30	15
8	NCYC 2592	35	20

### Strain screening using Phenotypic Microarray

**Fig 2:**
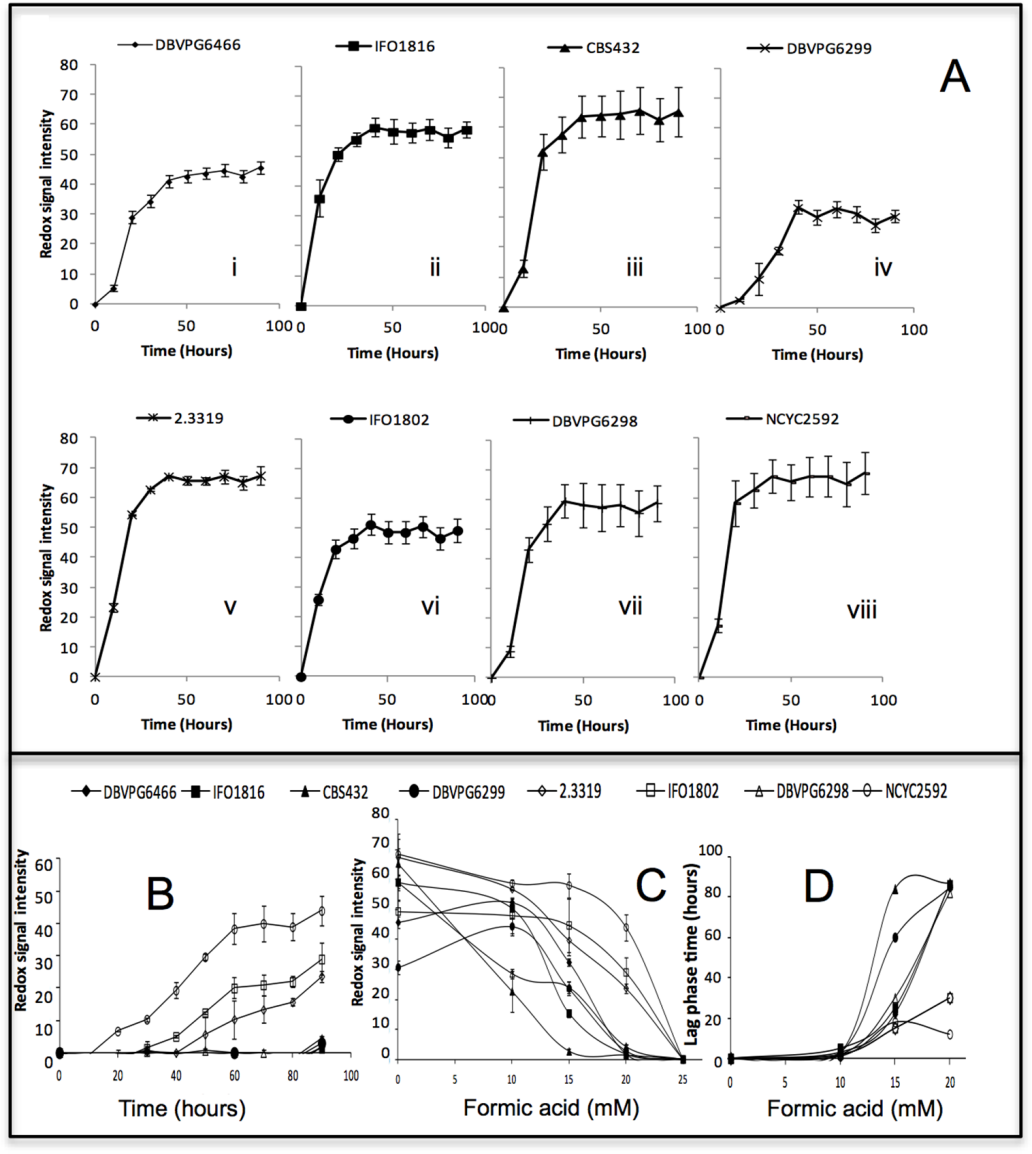
Metabolic output profiles of *Saccharomyces* spp on media containing formic acid (0–20 mM) incubated at 30°C under anaerobic conditions. (A) Control (0 mM) media without formic acid. (B) Formic acid 20 media. (C) Duration of lag phase in PM at formic acid 0,10,15 and 20 mM media. (D) Maximum redox signal intensity after 90 hours at formic acid 0, 10, 15, 20 and 25 mM media. Error bars represent the standard deviation of 3 replicates (Biolog unit = redox signal intensity).

Phenotypic microarray was used to investigate the effect of formic acid on yeast metabolic output, defined here as redox signal intensity [23] (Fig 2). The presence of formic acid elicited a concentration-dependent reduction in redox signal intensity (Fig 2D). *S*. *cerevisiae* NCYC 2592, *S*. *paradoxus* DBVPG6466, *S*. *kudriavzeii* IFO1802 and *S*. *arboricolus* 2.3319 demonstrated their capacity to tolerate 10 and 15 mM formic acid as high redox signal intensity were shown in phenotypic microarray; while the sensitive strains to these concentrations were *S*. *paradoxus* CBS432 and *S*. *bayanus* DBVPG6299. Increase in formic acid to 20 mM exerted more inhibitory effects on all the yeast strains (Fig 2B) and reduced the glucose utilization as compared with the control. The redox signal intensity of strains *S*. *kudriavzeii* IFO1802, *S*. *arboricolus* 2.3319 and *S*. *cerevisiae* NCYC 2592 indicated that these stains tolerate 20 mM formic acid, although both lag phase and maximum redox signal intensity were affected (Fig 2C and 2D). At 25 mM formic acid, there was no metabolic output observed, showing none of the strains could tolerate 25 mM formic acid in phenotypic microarray experiments.

### The formic acid tolerance of *S*. *arboricolus* strains during fermentation

**Fig 3:**
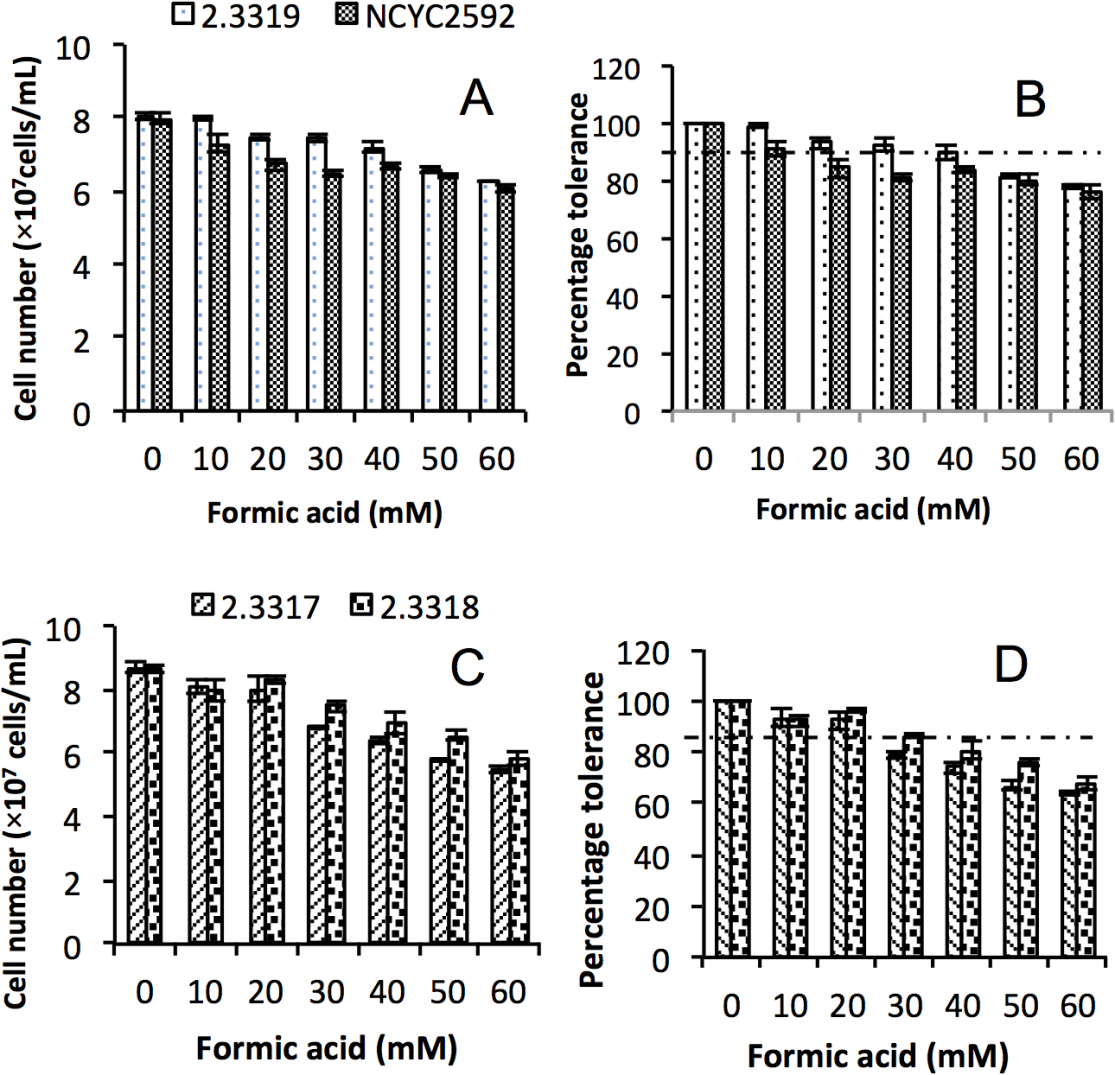
Growth profile (A and C) and percentage tolerance (B and D) of *Saccharomyces arboricolus* 2.3317, 2.3318 and 2.3319 and *Saccharomyces cerevisiae* NCYC2592 in the presence of formic acid (0–60 mM) using YPD medium. Values are the mean of three experiments and vertical error bars represent standard deviation.

According to the spot plate and phenotypic microarray screening experiments, strain *S*. *kudriavzeii* IFO1802 and *S*. *arboricolus* 2.3319 showed higher formic acid tolerance than other strains screened in this study. *S*. *arboricolus* 2.3319 was chosen for further study based on the genome sequence variability among the *Saccharomyces* spp (formerly known as *Saccharomyces* sensu strict yeast) as related to *S*. *cerevisiae* and as novel *Saccharomyces* species isolated from Tree bark [19, 24, 25]; also the availability of two other *S*. *arboricolus* (2.3317 and 2.3318) for assessment of formic acid tolerance in our lab. It was reported that *S*. *arboricolus* 2.3317 and *S*. *arboricolus* 2.3318 shared 100% similarity based on their multigene analysis [26]. In these fermentation experiments, pH was controlled at 4.5 by buffering. Therefore, higher formic acid concentrations (up to 60 mM) were investigated. *S*. *arboricolus* 2.3319 was firstly assessed for formic acid tolerance during fermentation and the results were compared with the reference strain *S*. *cerevisiae* NCYC2592 (Fig 3A and 3B). *S*. *arboricolus* 2.3319 and *S*. *cerevisiae* NCYC2592 strains showed the highest cells number of 8.58 x 10^7^ cells/mL and 8.07 x 10^7^ cells/mL respectively when cultured in the control medium (without formic acid addition). Increase in formic acid concentrations retarded yeast growth where at 60 mM cells number of 6.25± 0.03 × 10^7^ cells/mL and 6.03± 0.10 x 10^7^ cells/mL for *S*. *arboricolus* 2.3319 and *S*. *cerevisiae* NCYC2592, were obtained respectively. In fermentations containing formic acid at 10–40 mM, *S*. *arboricolus* 2.3319 maintained over 90% of its relative cell growth in comparison to the control, while the relative cell growth of *S*. *cerevisiae* NCYC2592 dropped below 90% in fermentations in the presence of 20 mM or higher concentrations of formic acid. These results showed that *S*. *arboricolus* 2.3319 has higher formic acid tolerance than *S*. *cerevisiae* NCYC2592. Based on these results, *S*. *arboricolus* 2.3317 and 2.3318 were further assessed for the formic acid tolerance with the hypothesis that the strains from the same species may share similar weak acid tolerance properties. As shown in Fig 3C and 3D, strain *S*. *arboricolus* 2.3317 and 2.3318 exhibited good formic acid tolerance (relative cell growth is over 90%) at concentrations of 10 and 20 mM. However, when the formic acid concentration increased to 30 mM or higher, cell growth of strains *S*. *arboricolu* 2.3317 and 2.3318 was affected.

During fermentations in the presence of 40 mM formic acid *S*. *arboricolu*s 2.3318, 2.3319 and *S*. *cerevisiae* NCYC2592 all showed improved ethanol production (Table 3), however, further increase formic acid concentration to 50 or 60 mM led to a decrease in ethanol synthesis for all strains. *S*. *arboricolus* 2.3319 demonstrated similar ethanol fermentation capacity in terms of final ethanol concentration and ethanol yield as *S*. *cerevisiae* NCYC2592. This indicated that *S*. *arboricolus* strain 2.3319 could be a good candidate for bioethanol fermentations.

**Table 3:** Ethanol concentration and yield summary from *Saccharomyces* spp fermentation of glucose in the presence of formic acid. Data are the mean of triplicate experiments and standard deviation.

Formic acid (mM)	*S*. *arboricolus* 2.3317		*S*. *arboricolus* 2.3318		*S*. *arboricolus* 2.3319		*S*. *cerevisiae* NCYC2592	
	Ethanol concentration (g/l)	Ethanol yield	Ethanol concentration (g/l)	Ethanol yield	Ethanol concentration (g/l)	Ethanol Yield	Ethanol concentration (g/l)	Ethanol yield
0	8.54±0.15	0.43±0.01	8.92±0.46	0.45±0.02	9.61±0.09	0.48±0.00	9.80±0.05	0.49±0.00
10	8.48±0.67	0.42±0.03	8.97±0.16	0.45±0.01	9.54±0.08	0.48±0.00	9.50±0.16	0.48±0.01
20	8.48±0.20	0.42±0.01	8.92±0.80	0.45±0.09	9.48±0.22	0.47±0.01	9.62±0.11	0.48±0.01
30	8.38±0.45	0.42±0.02	9.20±0.28	0.46±0.01	9.95±0.47	0.50±0.02	9.92±0.42	0.50±0.02
40	8.53±0.10	0.43±0.01	9.03±0.33	0.45±0.02	9.91±0.12	0.50±0.01	10.20±0.36	0.51±0.02
50	8.50±0.04	0.42±0.00	8.81±0.44	0.44±0.02	9.89±0.11	0.49±0.01	10.04±0.31	0.50±0.02
60	8.41±0.03	0.42±0.00	8.96±0.05	0.45±0.00	9.13±0.45	0.47±0.02	8.89±0.11	0.44±0.01

**Fig 4:**
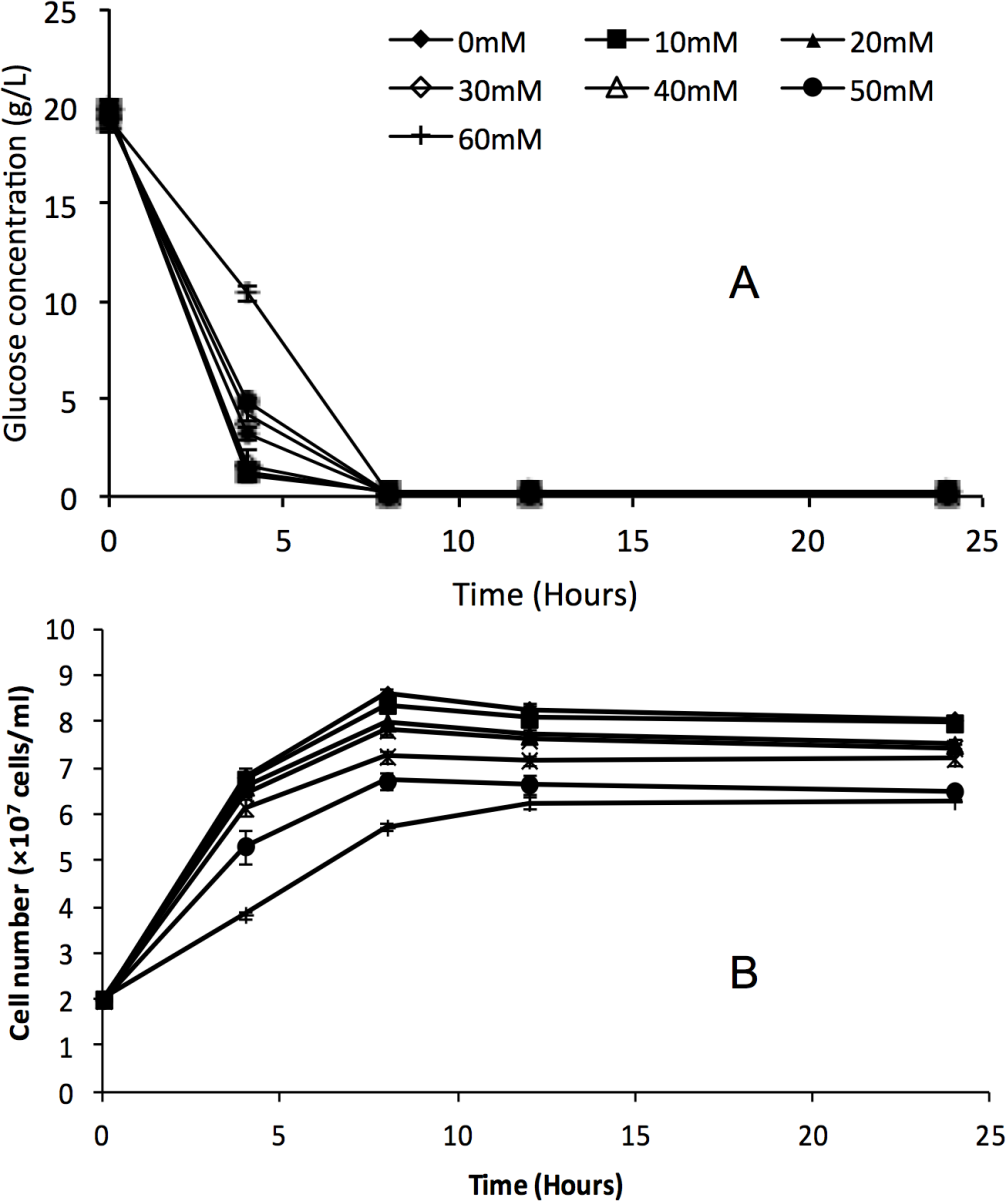
Time course profiles of glucose consumption (A) and cell growth curve (B) of *Saccharomyces arboricolus* 2.3319 in fermentation using YPD medium and formic acid (0–60 mM). Values are the mean of three experiments and vertical error bars represent standard deviation.

**Fig 5:**
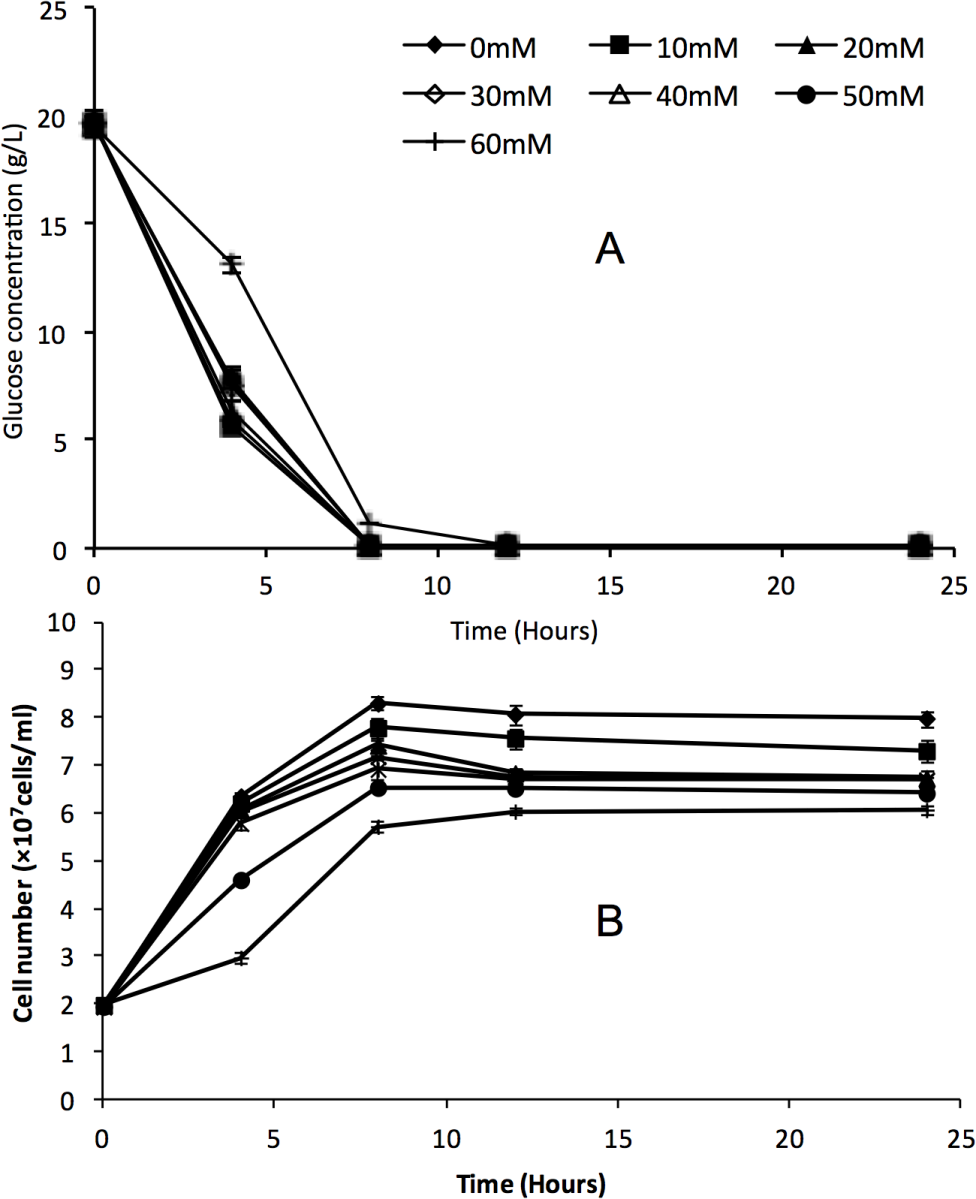
Time course profiles of glucose consumption (A) and cell growth curve (B) of *Saccharomyces cerevisiae* NCYC 2592 in fermentation using YPD medium and formic acid (0–60 mM). Values are the mean of three experiments and vertical error bars represent standard deviation.

**Fig 6:**
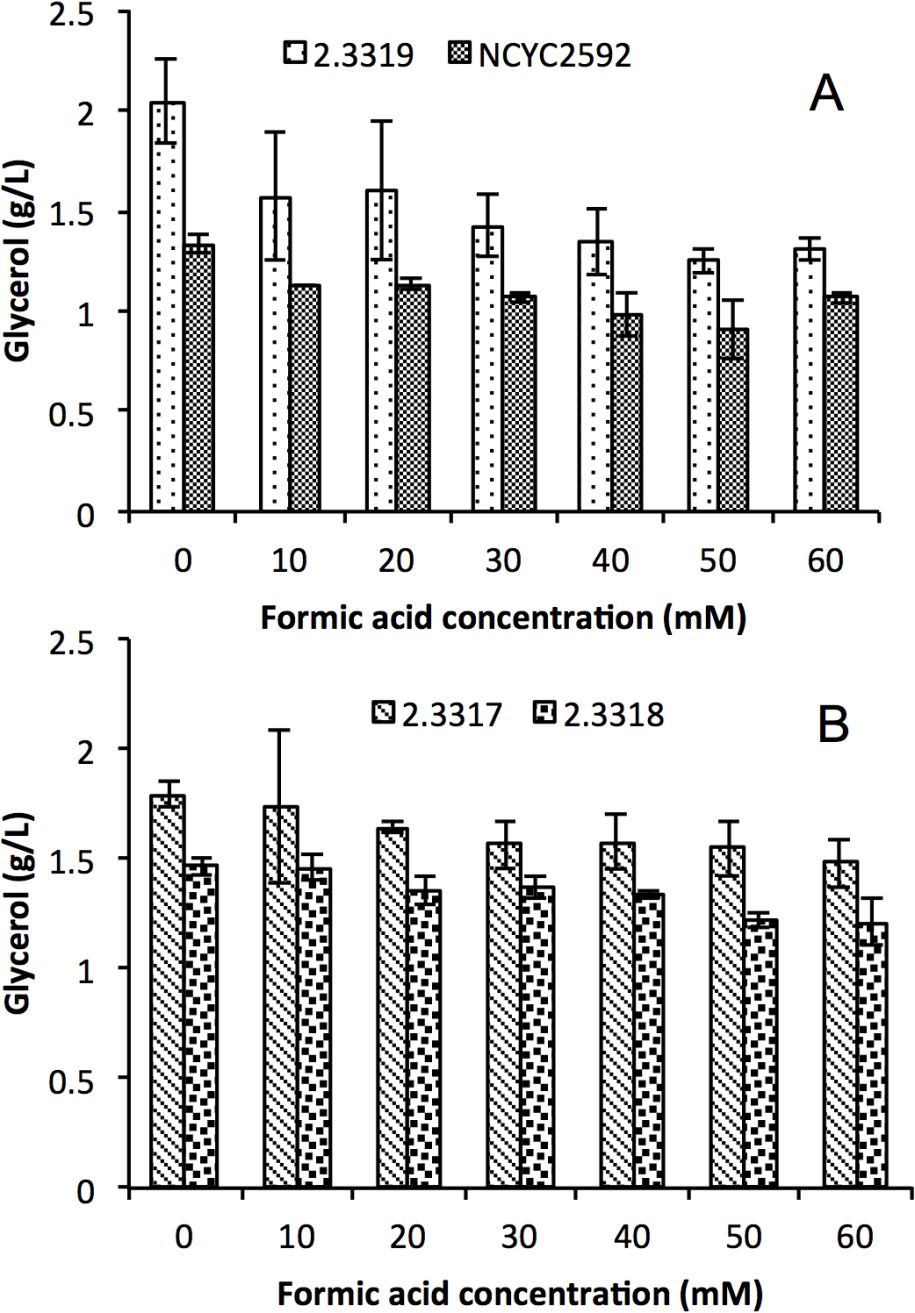
Level of glycerol produced by *Saccharomyces cerevisiae* NCYC 2592 and *Saccharomyces arboricolus* 2.3319 (A), and *Saccharomyces arboricolus* 2.3317 and *Saccharomyces arboricolus* 2.3318 (B) in the presence of formic acid (0–60 mM) using YPD medium. Fermentation was carried out under anaerobic condition, incubated at 30°C and samples were analyzed after 24 hours. Values are the mean of three experiments and vertical error bars represent standard deviation.

In order to further explore the response of yeast to formic acid, glucose utilization, glycerol production and glycogen production of *S*. *arboricolu*s 2.3319 and *S*. *cerevisiae* NCYC2592 were determined. There was no significant difference in glucose consumption between *S*. *arboricolus* 2.3319 (Fig 4A) and *S*. *cerevisiae* NCYC2592 at all concentrations (Fig 5A), although both strains consumed glucose faster in fermentations with a lower initial formic acid concentration. These results agreed with the cell growth curves observed in these fermentations (Figs 4B and 5B). Strains in fermentations with a higher formic acid concentration grew slower and ended at a relatively lower final cell concentration (Figs 3A, 3C, 4B and 5B). Compared with *S*. *cerevisiae* NCYC2592, *S*. *arboricolus* 2.3319 consumed glucose faster at the 4-hour data point. Glycerol is generally considered to be associated with stress tolerance [27]. At all formic acid concentrations, *S*. *arboricolus* 2.3319 produced more glycerol than *S*. *cerevisiae* NCYC2592 (Fig 6A). In the fermentations with 40 mM formic acid, *S*. *arboricolus* 2.3319 produced 1.37 ± 0.16 g/L glycerol while *S*. *cerevisiae* NCYC2592 produced 1.02 ± 0.11 g/L glycerol. Compared within *S*. *arboricolus* strains, glycerol produced by *S*. *arboricolus* 2.3317 and *S*. *arboricolus* 2.3318 was similar to *S*. *arboricolus* 2.3319 (Fig 6).

**Fig 7:**
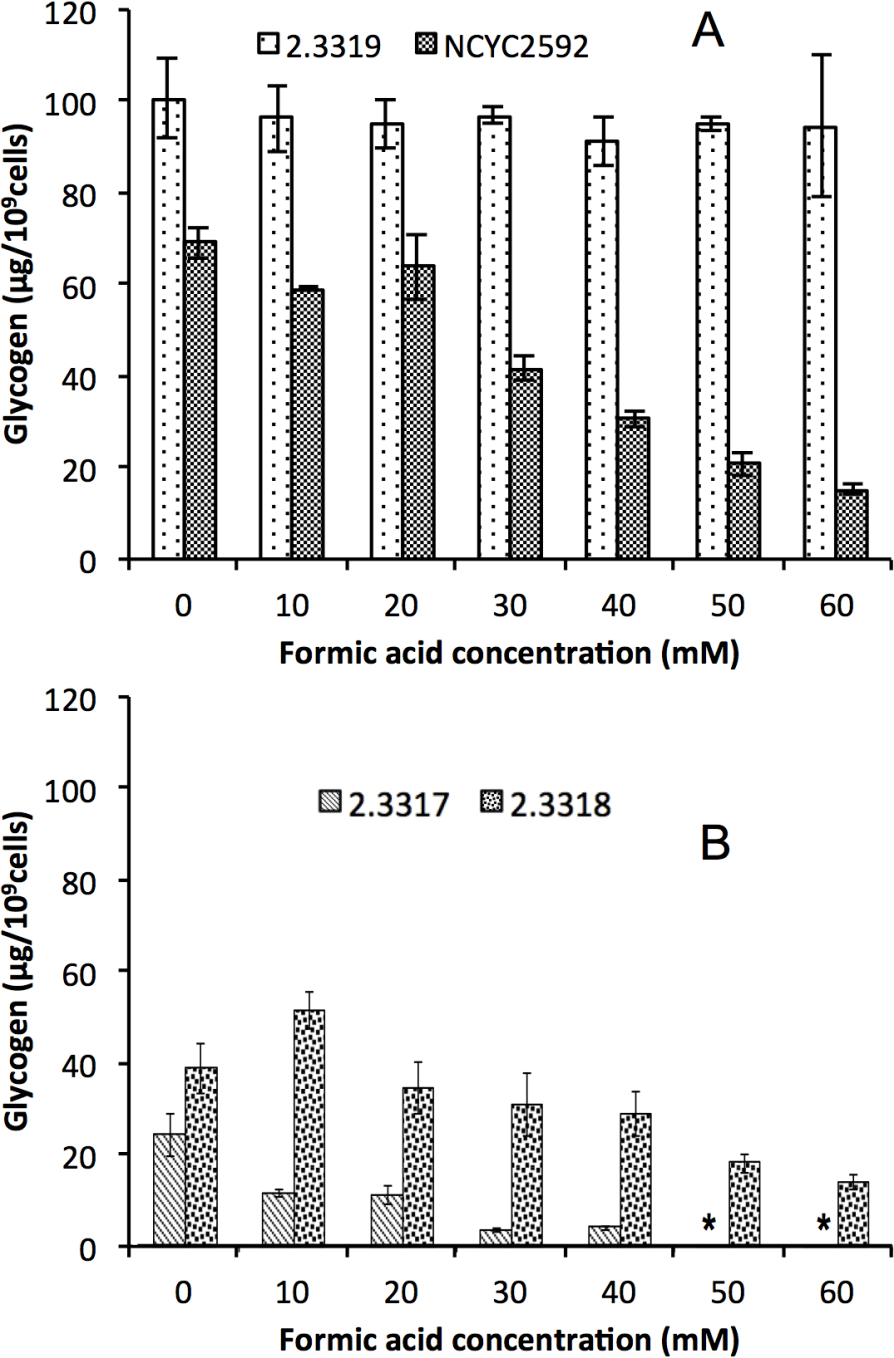
Intracellular glycogen accumulation by *Saccharomyces cerevisiae* NCYC 2592 and *Saccharomyces arboricolus* 2.3319 (A), and *Saccharomyces arboricolus* 2.3317 and *Saccharomyces arboricolus* 2.3318 (B) in the presence of formic acid (0–60 mM) using YPD medium. Fermentation was carried out under anaerobic condition, incubated at 30°C and samples were analyzed after 24 hours. Values are the mean of three experiments and vertical error bars represent standard deviation. *- Data are not available.

Accumulation of intracellular glycogen in yeast cells exposed to formic acid stress during fermentation was also determined. Higher accumulation of glycogen in yeast cells may act as an energy reserve maintaining cell viability when stressed [28]. Fig 7 revealed that increase in formic acid concentration decreased intracellular glycogen accumulation in all strains. In comparison, *S*. *arboricolus* 2.3319 accumulated significantly higher concentrations of glycogen than *S*. *cerevisiae* NCYC2592 (Fig 7A), and *S*. *arboricolus* 2.3317 and *S*. *arboricolus* 2.3318 (Fig 7B) in formic acid media from 0 to 60 mM, with the maximum intracellular glycogen concentration of 100.64 ± 8.82 μg/10^9^ cells. This higher accumulation of intracellular glycogen confirmed *S*. *arboricolus* 2.3319 to be more formic acid tolerant than *S*. *arboricolus* 2.3317, 2.3318 and *S*. *cerevisiae* NCYC2592.

## Discussion

The hydrolysates of lignocellulosic substrates can contain toxic compounds which are released during pretreatment. These compounds can negatively affect the fermentation efficiency of *Saccharomyces cerevisiae* strains. Consequently, some form of strain- or process-adaptation is required to prevent the detrimental impact of inhibitors. Although the composition of toxic compounds differs among lignocellulosic biomass and pretreatment methods, it has been widely reported that weak acids such as formic acid are produced via a range of pre-treatment methods, including the use of dilute sulfuric acid and hot-compressed water treatments [12, 29]. Hasunuma *et al*. [30] reported that 10–20 mM concentrations of formic acid in the pretreated biomass hydrolysate were sufficient to hinder fermentation by *Saccharomyces cerevisiae*. Thus screening to identify yeast strains which are tolerant to formic acid may help to improve the efficiency of lignocellulosic ethanol production.

Yeast, particularly *Saccharomyces cerevisiae* strains had been screened for the tolerance of inhibitors in the lignocellulosic hydrolysate as well as osmotic, heat and acid stresses [31, 32]. However, screening of non-*Saccharomyces cerevisiae* strains for inhibitor tolerance, particularly formic acid has been limited. It has been shown that weak acids inhibit yeast cells through similar inhibitory mechanisms [33]. Therefore, screening of formic acid tolerance strains using spot plate and phenotypic microarray could identify yeasts with potential tolerance to other weak acids as well, especially as formic acid is considered to be more toxic than acetic acid and levulinic acid [12, 30]. The exploration of wild *Saccharomyces* sp. for inhibitor tolerance could lead to the discovery of novel yeast strains with distinct genetic background associated with formic acid tolerant. The resulting high tolerant strains can be mated with *Saccharomyces cerevisiae* to form hybrid diploids that will utilize hydrolysate for bioethanol production [19, 30].

In this report, we examined the formic acid tolerance of 7 non- *Saccharomyces cerevisiae* strains. YPD and YNB agar media were firstly employed with the addition of different concentrations of formic acid. With increasing formic acid concentration growth variation was observed amongst the strains. Strains *S*. *paradoxus* DBVPG6466, *S*. *kudriavzeii* IFO1802, *S*. *arboricolus* 2.3319 and *S*. *cerevisiae* NCYC2592 exhibited tolerance to 35 mM and 20 mM formic acid on YPD and YNB media respectively, while other strains did not grow. The tolerance levels of yeast strains to formic acid concentration in YPD was higher than tolerance in YNB because YNB as minimal medium affected cell growth [34]. The spot plate method has also been used by other researchers for the study of inhibitory effect on yeast strains caused by weak organic acids [35].

Screening of yeast strains for stress and inhibitor tolerance is an important step in lignocellulosic bioethanol production. The utilization of only spot plate analysis for the identification of tolerance strains is easy to operate and has less equipment dependent, but is slow and time consuming [8]. Phenotypic Microarray (PM) is an integrated system for high-throughput screening of microorganisms. Compared with spot plate method, PM is a quick, automatic and liquid-culture based technique that allows screening of a large number of strains at the same time under various conditions [8, 23]. However, it relies on the measurement of cell respiratory activity rather than cell growth [8, 23]. PM has recently been used to characterize yeast tolerance to various stresses, e.g. temperature, ethanol and inhibitors in the lignocellulosic hydrolysate [19]. Seven non-*Saccharomyces cerevisiae* strains were screened using the phenotypic microarray. The results demonstrated that *S*. *arboricolus* 2.3319 and *S*. *kudriavzeii* IFO1802 could tolerate 20 mM formic acid (Fig 2). This agreed with results obtained using YNB spot plate method, as PM uses a minimal medium as well. Strain *S*. *paradoxus* DBVPG6466 could not tolerate 20 mM formic acid in PM experiments, suggesting that strains may have different acid tolerance abilities between solid culture (spot plate) and liquid culture (PM). This indicated that PM is a good complement to the traditional spot plate screening method.

Based on the above results, *S*. *arboricolus* 2.3319 and two other *S*. *arboricolus* (2.3317 and 2.3318) strains were selected and tested for formic acid tolerance and bioethanol production at a laboratory scale. Compared with lab strain, *S*. *cerevisiae* NCYC2592, *S*. *arboricolus* 2.3319 maintained over 90% relative cell growth in the presence of formic acid (10 to 40 mM) (Fig 3). The reference strain, *S*. *cerevisiae* NCYC2592 also showed good formic acid tolerance, but the relative cell growth reduced to 90% or lower when formic acid concentration was 20 mM or higher. In all cases, the addition of formic acid decreased cell number (Fig 3). The exact reason for this reduction is unknown. A potential reason may be due to the diversion of energy (ATP) to pump out protons at the expense of cell biomass production [36]. Similar results were reported by Huang *et al*. [37] in the investigation of the inhibitory effect on *S*. *cerevisiae*. In comparison with *S*. *arboricolus* 2.3319, *S*. *arboricolus* 2.3317 and 2.3318 did not exhibited high formic acid tolerance, showing no correlation between species tolerance to formic acid. It was also established by Almeida *et al*. [31] who screened strains of *S*. *cerevisiae* and found out that inhibitor tolerance was strain specific and not species specific.

The formic acid has both positive and negative effects on bioethanol fermentation. At low or medium acid concentration (e.g. 30–40 mM), the presence of formic acid increased both ethanol yield and titer in both *S*. *arboricolus* 2.3319 and *S*. *cerevisiae* NCYC2592 strains (Table 3). A possible explanation is that glycolytic activity may be increased in order to produce more ATP required to pump protons out of the cells at the expense of cell biomass, while ethanol production was increased for ATP production [15, 30, 38]. This investigation supports the work of Teherzadeh *et al*. [39], who reported that during fermentation, exposure of *S*. *cerevisiae* to acetic acid (< 25 m) stimulated ATP production and increased the rate of ethanol production when compared to an unstressed control. Further increase in formic acid concentration to 60 mM led to drops in ethanol production (Table 3). The increased toxicity was associated with formic acid’s high plasma membrane permeability [12, 30, 37]. But formic acid did not affect glucose consumption in either *S*. *arboricolus* or *S*. *cerevisiae* strains (Fig 4). Similar result was reported in yeast fermentations with acetic acid [34]. Compared with fermentations using *S*. *cerevisiae* NCYC2592, fermentations using *S*. *arboricolus* 2.3319 resulted in similar or higher ethanol yields, which were also very close to the theory glucose to ethanol yield of 0.51 (Table 3). These ethanol yields were also higher than or similar to several reports in fermentations using other high tolerance ethanol producing strains, e.g. *S*. *cerevisiae* YZ1 [31], *S*. *cerevisiae* Y-1528 [32] and an isolate of Bekonang [34].

In the fermentation process, *S*. *arboricolus* 2.3319 contains inherently higher glycerol concentrations under control and formic acid stress than *S*. *cerevisiae* NCYC2592 (Fig 6). This may be of benefit for *S*. *arboricolus* to tolerate formic acid than that of strain *S*. *cerevisiae* NCYC2592 though further experimentation would be required to establish if glycerol could be acting as a polyol to the cells [27]. The investigation agreed with the work of Tomas-Pejo *et al*. [40] that higher glycerol production resulted to strain tolerance to lignocellulosic hydrolysate inhibitors which is an indication of better cell growth. Lower amounts of glycerol were produced when formic acid addition was increased. The low glycerol formation may be attributed to the re-oxidation of NADH to NAD^+^ for the ATP formation [41]. Taherzadeh *et al*. [42] also reported that the addition of acetate resulted in a decrease in glycerol production. Intracellular glycogen has been considered as an important reserved carbohydrate in the survival of yeasts when yeasts were exposed to stress [43, 44]. In this study, *S*. *arboricolus* 2.3319 accumulated more intracellular glycogen than *S*. *cerevisiae* NCYC2592 (Fig 7), which may help the strain to tolerate high formic acid concentrations. The intracellular glycogen may be playing a dual role according to Deshpande *et al*. [28], by providing energy and carbon skeleton required for cell growth as well as minimize leakage through plasma membrane by the stressful effect of formic acid. This agreed with Somani *et al*. [45] that higher content of glycogen favored the survival of yeast strain during environmental stress conditions.

## Conclusion

This study reported a simple and fast method for screening non-*S*. *cerevisiae* strains for formic acid tolerance. In comparison to other non-*S*. *cerevisiae* strains, *S*. *arboricolus* 2.3319 was shown to be tolerant to formic acid using both spot plate and PM techniques, which was then confirmed by a series of fermentations. Fermentation experiments demonstrated that *S*. *arboricolus* 2.3319 produced similar amounts of ethanol to the reference strain *S*. *cerevisiae* NCYC2592, indicating its potential to be used as a novel bioethanol producer or as a source of gene donor to other higher ethanol producing strains that are inhibitor-sensitive. *S*. *arboricolus* 2.3319 produced more glycerol and glycogen than the reference strain, which may enable its good formic acid tolerance ability.
